# Comprehensive Analysis of Long Non-coding RNA Modulates Axillary Bud Development in Tobacco (*Nicotiana tabacum* L.)

**DOI:** 10.3389/fpls.2022.809435

**Published:** 2022-02-14

**Authors:** Lin Wang, Junping Gao, Chen Wang, Yalong Xu, Xiaoxu Li, Jun Yang, Kai Chen, Yile Kang, Yaofu Wang, Peijian Cao, Xiaodong Xie

**Affiliations:** ^1^China Tobacco Gene Research Center, Zhengzhou Tobacco Research Institute of China National Tobacco Corporation (CNTC), Zhengzhou, China; ^2^China Tobacco Hunan Industrial Co., Ltd., Changsha, China

**Keywords:** long non-coding RNAs, axillary bud, plant hormone, glycometabolism, topping, tobacco

## Abstract

Long non-coding RNAs (lncRNAs) regulate gene expression and are crucial for plant growth and development. However, the mechanisms underlying the effects of activated lncRNAs on axillary bud development remain largely unknown. By lncRNA transcriptomes of axillary buds in topped and untopped tobacco plants, we identified a total of 13,694 lncRNAs. LncRNA analysis indicated that the promoted growth of axillary bud by topping might be partially ascribed to the genes related to hormone signal transduction and glycometabolism, *trans*-regulated by differentially expressed lncRNAs, such as MSTRG.52498.1, MSTRG.60026.1, MSTRG.17770.1, and MSTRG.32431.1. Metabolite profiling indicated that auxin, abscisic acid and gibberellin were decreased in axillary buds of topped tobacco lines, while cytokinin was increased, consistent with the expression levels of related lncRNAs. MSTRG.52498.1, MSTRG.60026.1, MSTRG.17770.1, and MSTRG.32431.1 were shown to be influenced by hormones and sucrose treatments, and were associated with changes of axillary bud growth in the overexpression of *NtCCD8* plants (with reduced axillary buds) and RNA interference of *NtTB1* plants (with increased axillary buds). Moreover, MSTRG.28151.1 was identified as the antisense lncRNA of *NtTB1.* Silencing of MSTRG.28151.1 in tobacco significantly attenuated the expression of *NtTB1* and resulted in larger axillary buds, suggesting the vital function of MSTRG.28151.1 axillary bud developmen by *NtTB1*. Our findings shed light on lncRNA-mRNA interactions and their functional roles in axillary bud growth, which would improve our understanding of lncRNAs as important regulators of axillary bud development and plant architecture.

## Introduction

Long non-coding RNAs (lncRNAs) are RNAs longer than 200 bp and lack encoding capability (Kapranov et al., 2007; Dinger et al., 2009). Plant lncRNAs can play roles at the transcriptional, post-transcriptional, and epigenetic levels, and are characterized by epigenetic markers, developmental stages, and tissue-specific expression (Zhang et al., 2013). LncRNAs have been systematically identified in some model plants. Transcriptome sequencing has led to the identification of 6,480 lncRNAs related to biological and abiotic stresses in *Arabidopsis thaliana* (Liu et al., 2012), 2,224 lncRNAs in rice (Zhang et al., 2014), and 2,542 drought-responsive lncRNAs in poplar (Shuai et al., 2014). Plant lncRNAs are involved in the regulation of plant flowering, stress response, reproductive growth, and other vegetation processes. The lncRNA, *FLORE*, is involved in circadian rhythm regulation and affects plant flowering during vernalization (Henriques et al., 2017). Two lncRNAs, *COOLAIR* and *COLDAIR*, affect *Arabidopsis* flowering by regulating the expression of flowering locus C (Yamaguchi and Abe, 2012). Plant growth and metabolism are also influenced by the lncRNA, *IPS1*, which has a substantial impact on phosphorus uptake and phosphorus use efficiency (Huang et al., 2011). Moreover, *T5120*, is a crucial regulator of nitrate response and assimilation in *Arabidopsis* (Liu F. et al., 2019), and lncRNA1459 inhibits ethylene synthesis and lycopene accumulation in tobacco (Li et al., 2018). LncRNAs also regulate stress response in plants. For example, lncRNA354 plays an important role in regulating salt tolerance in upland cotton (Zhang et al., 2021), and genome-wide analysis has revealed the role of lncRNAs in regulating heat tolerance in Chinese cabbage (Song et al., 2021).

The branching pattern of a plant is regulated by the outgrowth of axillary meristems, and improved branching patterns lead to improved crop yield, quality and increased value (Schmitz and Theres, 2005). Plant hormones can regulate the formation of axillary meristems, activate dormant axillary buds, and regulate the growth of axillary buds (Zhuang et al., 2019). For example, auxins inhibit the development of branches by down-regulating the polar transport of PIN proteins (Xie et al., 2017), and gibberellins inhibit the formation of axillary buds by controlling DELLA-SPL9 complex activity (Zhang et al., 2020). In contrast to auxins, cytokinins (CTK) promote the growth of lateral buds (Waldie and Leyser, 2018). Strigolactones (SLs) are newly identified plant hormones that are primarily produced in the root and interact with other hormones to inhibit the development of axillary buds (Crawford et al., 2010; Dun et al., 2012). *CCD8* (carotenoid cleavage dioxygenase 8) gene encoding to CCD enzymes, involved in the synthesis of SLs (Jia et al., 2018). CRISPR/Cas9-mediated mutagenesis of *NtCCD8* has been shown to promote axillary bud growth (Gao et al., 2018). Recent studies have suggested that sugars are the initial trigger for hormonal networks that control bud outgrowth (Schneider et al., 2019). Some analogs of sucrose, when transported to the axillary buds, also enable the activation of dormant axillary buds (Bertheloot et al., 2020; Wang M. et al., 2021). In addition, trehalose 6-phosphate has been found to participate in the development of axillary bud outgrowth (Fichtner et al., 2017). As the downstream of these signals, transcription factors (TFs) could regulate axillary bud development. For example, Teosinte Branched1 (TB1), a TCP transcription factor, is an integrator of hormonal, nutritional, and environmental signals that inhibit lateral bud growth (Aguilar-Martínez et al., 2007; Dong et al., 2019). The overexpression of *TB1* inhibits axillary bud formation after decapitation in tobacco (Ding et al., 2020). Moreover, target genes corresponding to lncRNAs have been reported to participate in the signaling pathways of plant hormones such as gibberellin (GA), ethylene, and jasmonic acid (Xiao et al., 2020). However, studies on the regulation of axillary bud development by lncRNAs in plants are still incomplete.

Apical dominance, a phenomenon in which terminal bud growth is promoted and lateral branch growth is inhibited, occurs in many plants (Cline and Sadeski, 2002). Loss of the terminal bud leads to lateral branch formation and rapid growth (Taylor et al., 2008). Tobacco (*Nicotiana tabacum*) is a classic cash crop that is affected by the development of axillary buds, and intervention by topping switches tobacco plants from vegetative to reproductive growth. Following topping, the axillary buds located in the axils of tobacco leaves grow rapidly and form a large number of lateral branches, thus consuming a large number of nutrients (Zhao et al., 2018). Studies investigating the effects of topping on the composition and function of RNAs in tobacco have reported topping-responsive non-coding RNAs and proteins in roots and topping-induced mRNAs in axillary shoots (Qi et al., 2012; Li F. et al., 2016; Chen et al., 2019). However, to our knowledge, no studies have investigated the function of lncRNAs in axillary bud development. In this study, we used RNA sequencing (RNA-seq) to elucidate the molecular mechanisms of lncRNAs regulating axillary bud growth by comparing how axillary buds respond to topping at different times and locations. Furthermore, we constructed a lncRNA-mRNA co-expression network and predicted the antisense lncRNAs of genes associated with axillary bud development. Based on our results, we further investigated the potential functions of differentially expressed lncRNAs in response to axillary bud growth.

## Materials and Methods

### Plant Materials and Cultivation

*Nicotiana tabacum* K326 plants with a strong growth of axillary buds were grown in a greenhouse. Morphologically similar tobacco plants were selected for topping and their apical buds were removed. We collected 18 samples of tobacco axillary buds growing uniformly at different stages: upper axillary buds before topping (Cku), upper axillary buds at different time points after topping (3 h: 3 hu; 12 h: 12 hu; and 24 h: 24 hu), lower axillary buds before topping (Ckd), and lower axillary buds 3 h after topping (3 hd). The samples were packed in self-sealed bags, treated with liquid nitrogen, and stored in a freezer at −80°C for subsequent tests.

### Extraction and Detection of Plant Hormones

The samples taken from the ultra-low temperature freezer were ground into a powder. 10 μL of internal standard solution (100 ng/mL) and 1 mL methanol/water/formicacid were added to the powdered sample (50 mg). The mixture was scrolled and extracted by a refrigerated centrifuge at 12,000 r/min for 5 min. The concentrated solution was re-dissolved in 100 μl of methanol (80% v/v), using a 0.22 μM ultrafiltrate membrane for LC–MS/MS analysis (Floková et al., 2014; Li Y. et al., 2016). UPLC-ESI-MS/MSsystem (UPLC’ExionLC*™*AD, https://sciex.com.cn/; MS, Applied Biosystems 6500 Triple Quadrupole, https://sciex.com.cn/) was used for separation and quantification. The compounds were separated by C18 column (100 mm × 2.1 mmi.d’1.8 μm) and held at 40°C. The mobile phase consisted of (A) HPLC water (containing 0.04% acetic acid) and (B) acetonitrile (containing 0.04% acetic acid). The following gradient program was used in the procedure: 0–1 min, 5% B; 1–8 min, 95% B; 8–9 min, 95% B; 9.1–12 min, 5% B. The flow rate was maintained at 0.35 mL/min, and the injection volume was 2 μL (Cai et al., 2014; Niu et al., 2014). The ESI source operation parameters were set as follows: ion source temperature of ESI at 550°C; curtain gas pressure (CUR) at 35 psi; positive ion spray voltage (IS) at 5,500 V, negative ion spray voltage (IS) at −4,500 V. Plant hormones were quantified and analyzed using scheduled multiple reaction monitoring and multiquant 3.0.3 software (Pan et al., 2010; Cui et al., 2015; Šimura et al., 2018).

### Transcriptome Sequencing and Data Filtering

The axillary buds were ground into a powder for RNA extraction (Hu and Xiong, 2014). The concentration and quality of RNA were evaluated using a NanoDrop system and the Agilent 2100 platform (Agilent Technologies, Inc., Santa Clara, CA, United States). Ribosomal RNA was removed from the total RNA, and the resulting mRNA was randomly broken into short fragments. The cDNA was purified using the QiaQuick testing kit and eluted by adding EB buffer solution. The ends were repaired, poly (A)-tails were added, and the adapter was ligated. The target segment was recovered and used for PCR amplification to complete the library construction. Finally, 18 libraries were constructed for chain-specific sequencing. Clean reads were acquired by removing reads containing the adapter, low-quality data, and poly N sequences. The Q20, Q30, and GC contents were retrieved from the cleaned data (Pertea et al., 2015; Kim et al., 2019).

### Annotation and Quantification of Long Non-coding RNAs

The clean data were compared with the reference genome using the read aligner HIAST2 (Kim et al., 2015). The assembled transcript isoforms were aligned to the annotation information of the reference genome. Using the CPC software program and the PLEK database (Kong et al., 2007; Finn et al., 2016), we identified lncRNA as transcripts longer than 200 bp on an open reading frame (ORF), their length being longer than 120 amino acids, with no potential encoding capability. The fragments per kilobase million (FPKM) of lncRNAs and mRNAs were calculated using Cuffdiff (version 2.1.1). Significantly differentially expressed lncRNAs and mRNAs were established as those with a *q*-value ≤ 0.05 and log_2_ (FPKM) ratio ≥ 1.5 using the Cufflinks software.

### Interaction Network Construction and Enrichment Analysis of Long Non-coding RNAs-mRNA Pairs

LncRNAs were predicted to influence axillary buds through the *trans*-regulation of mRNA expression. We used the expression levels of lncRNAs and mRNAs to calculate the correlation coefficients of lncRNA-mRNA pairs and screen the lncRNA-mRNA regulatory relationship (Wang R. et al., 2018). For the interactions to be considered as *trans*-regulation, we ensured that the *P*-value of the correlation coefficient was < 0.05 and the absolute value of the Pearson correlation coefficient was > 0.9. A lncRNA-mRNA co-expression network was built using our data. The lncRNAs were used as the central node, and the interaction network diagram was constructed using Cytoscape to visualize the regulatory network of lncRNAs and mRNAs.

To infer the functions of the lncRNAs, we conducted KEGG enrichment analysis of the target genes regulated by differentially expressed lncRNAs through the KOBAS software. Pathways with a corrected *P*-value of < 0.05 were considered significantly enriched. To further study axillary bud development, we used significantly differentially expressed mRNA and lncRNA (log_2_ (fold change) > 1.5) in the plant hormone signal transduction, glycometabolism pathways and transcription factors.

### Quantitative Real-Time PCR Analysis

Six critical lncRNAs and targeted genes were selected and used for validation by quantitative real-time PCR (qRT-PCR). RNA was isolated using a plant RNA rapid extraction kit (Genepure Plus, Imagene, China). The sample was converted to cDNA through RT-PCR with a reverse transcription kit (Takara Bio Inc., Shiga, Japan). The qPCR primers were designed by Primer 5.0 (Supplementary Table 1). qRT-PCR was conducted in a total reaction volume of 15 μl, including 1 μl of specific primers (10 μM), 7.5 μl of the SYBR Green qPCR mix, 2 μl of the template cDNA (50 ng/μL), and 4.5 μl of dH_2_O. The reactants were mixed before the PCR. The GAPDH gene was used as the internal control, and qPCR analysis was performed using a CFX96TM Real-Time PCR Detection System (Bio-Rad, Hercules, CA, United States) following the manufacturer’s recommendations. The relative gene expression levels were evaluated using the 2^–△△Ct^ method. The above methods were also used to analyze the expression levels of all genes in the paper, and the primers used are listed in Supplementary Table 1.

### Hormone and Sucrose Treatments

Tobacco seeds (K326) were sown in MS medium and grown in an incubator. Tobacco seedlings with four leaves were selected and transplanted into Hoagland nutrient solution. Three days later, plant hormones—including indole-3-acetic acid (IAA; 10 μmol/L), gibberellic acid (GA, 10 μmol/L), cytokinins (CTK, 10 μmol/L), abscisic acid (ABA, 10 μmol/L) and sucrose (100 mmol/L) were added to the nutrient solution. Tobacco seedlings cultivated in Hoagland solution were used as controls. The expression levels of MSTRG.52498.1, MSTRG.60026.1, MSTRG.17770.1, and MSTRG.32431.1 were estimated in the different treatment groups. The primers used are listed in Supplementary Table 1.

### Construction and Transformation of NtCCD8, NtTB1, and MSTRG.28151.1

With the *NtCCD8* and *NtTB1* gene sequence cloned from tobacco and the sequence information of MSTRG.28151.1 in the transcriptome data, primer 5.0 software was used to design primers for specific amplification the *NtCCD8* full length cDNA and the positive, negative fragments of *NtTB1*, MSTRG.28151.1. PCR products were separated by agarose gel electrophoresis and the target bands were cut for DNA fragment recovery. The primers used were listed in Supplementary Table 1.

For *NtCCD8*-OE vector construction, plant expression plasmid pBI121 was digested by *Bam*HI and *Sac*I enzymes. The pBI121 vector carries the Kana resistance gene as an antibiotic selectable marker. The *NtCCD8* full length cDNA was inserted into the pBI121 plasmid, behind the CaMV 35S promoter, to replace the *GUS* gene in PBI121. The recombinant expression vector pBI121-*NtCCD8-*OE was obtained. For RNAi vectors construction, the interference fragment was amplified with the reverse fragment primer and cloned into the pCambia2301-KY-RNAi *via Bam*HI/*Xba*I digestion and ligation. The pCambia2301-KY-RNAi vector carries the Kana resistance gene as an antibiotic selectable marker. Competent *Escherichia coli* DH5-α cells were transformed with reaction products and positive clones were screened as the intermediate carrier. The interference fragment was amplified with the forward fragment primers and cloned into the intermediate carrier *via Kpn*I digestion and ligation. The interference vector of pCambia2301-*NtTB1*-RNAi and pCambia2301- MSTRG.28151.1-RNAi were obtained. These vectors were transformed into *Agrobacterium tumefaciens* LBA4404 competent cells (Liedschulte et al., 2017).

### Statistical Analyses

Data analysis was conducted using GraphPad prism 9 and Excel 2016. All treatments and sample collection in this study were performed with at least three repetitions, and the data collected were analyzed by one-way ANOVA and Tukey’s test. *Correlation is significant at the 0.05 level, **Correlation is significant at the 0.01 level. For each biological replicate, three plants with the same growth were selected to collect samples and mixed into one biological replicate. At least nine tobacco plants are required for three biological replicates.

## Results

### Phenotypes of Axillary Buds in Different Positions After Topping

**FIGURE 1:**
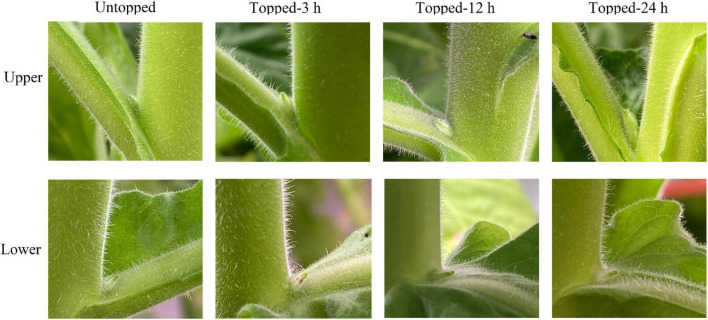
Upper and lower axillary buds development and phenotypes in untopped and topped tobacco plants. Upper axillary buds before topping (Cku), upper axillary buds at different time points after topping (3 h: 3 hu; 12 h: 12 hu; and 24 h: 24 hu), lower axillary buds before topping (Ckd), and lower axillary buds 3 h after topping (3 hd).

Topping has an important effect on the production and development of axillary buds in tobacco. We compared the growth and development of upper axillary buds in un-topped and topped tobacco plants at 3, 12, and 24 h after topping. The growth of the upper axillary buds was not obvious at the initial stage, however, their development was distinctly accelerated 24 h after topping. In addition, the lower axillary buds in the topping group showed little change at 3, 12, and 24 h, compared with the plants topping and untopped plants (Figure 1). These results indicated that the development of the upper axillary buds was influenced earlier in response to topping than the lower axillary buds. Due to the short time after the topping and the axillary bud developing rule from top to bottom, no observed changes in lower axillary bud within 24 h were normal phenomenon.

### RNA Sequencing Output and Characterization of Long Non-coding RNAs

We used RNA sequencing to determine the role of lncRNAs in axillary bud development in tobacco. The RNA quality of 18 samples (Cku, 3 hu, 12 hu, 24 hu, Ckd, and 3 hd; three biological replicates) was appraised and the samples were used for RNA sequencing. The clean reads, total number of mapped reads rate, and unique mapped reads are listed in Supplementary Table 2. The results indicated that the RNA-seq data were reliable for further analysis. The coding potential of transcripts was predicted using the RFAM, CPC, PLEK, and Swiss-Prot databases (Supplementary Figure 1A). The identified lncRNAs were classified as follows: 93.38% of the total lncRNAs were intergenic lncRNAs, 2.94% were intronic lncRNAs, and 3.67% were natural antisense *trans*-lncRNAs (Supplementary Figure 1B). For better comparison and exploration of the identified lncRNAs in tobacco, we compared the length, exon number, and ORF length of lncRNAs with those of mRNAs (Supplementary Figures 1C–E). The overall length, number of exons, and ORF lengths were longer for mRNAs than for lncRNAs. The expression levels of lncRNAs and mRNAs were calculated using FPKM values. In total, we identified 13,694 lncRNAs and 69,500 mRNAs (Supplementary Table 3). Compared with the expression of lncRNAs in tobacco plants before topping, the number of differentially expressed lncRNAs after topping was 64 (3 hu vs. Cku), 139 (12 hu vs. Cku), 136 (24 hu vs. Cku), and 20 (3 hd vs. Ckd) (Supplementary Figure 1F). In addition, we found 343 (3 hu vs. Cku), 878 (12 hu vs. Cku), 322 (24 hu vs. Cku), and 174 (3 hd vs. Ckd) genes that were differentially expressed between the groups (Supplementary Figure 1G). After topping, the number of lncRNAs and mRNAs detected in the upper axillary buds were the highest at 12 h, and were overall higher than those in the lower axillary buds.

### Interaction Network Construction of *Trans*-Regulated Protein-Coding Genes of Long Non-coding RNAs

**FIGURE 2:**
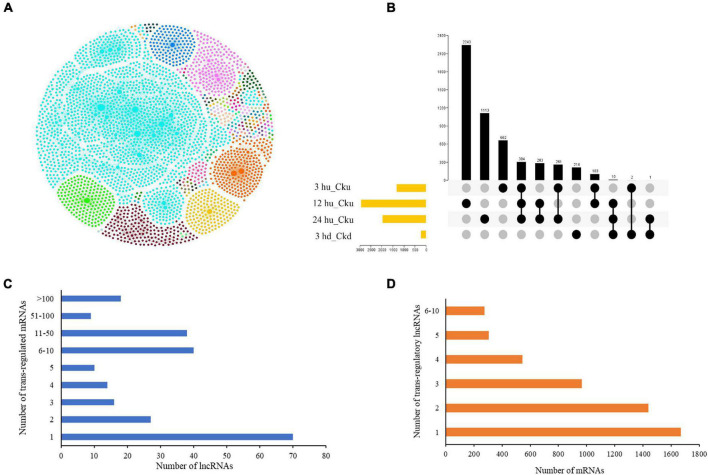
Interactions between lncRNAs and their *trans*-regulated mRNAs in tobacco plants. **(A)** The lncRNA-mRNA interaction network, including comparisons between all treatment groups (3 hu, 12 hu, 24 hu, and 3 hd vs. the respective control group). The nodes represent lncRNAs and mRNAs, and the edges represent the interactions between them. The node size corresponds to the number of interacting mRNAs or lncRNAs, and the edge color denotes the number of comparisons where different lncRNA-mRNA connections are present. **(B)** A Venn diagram displaying the common and specific matched lncRNA-mRNA pairs. **(C)** Number of differentially expressed mRNAs regulated by differentially expressed lncRNAs. **(D)** Number of differentially expressed lncRNAs regulating differentially expressed mRNAs.

LncRNAs have been found to regulate gene expression by interacting with promoters, enhancers, or other proteins that bind to these sites (Guil and Esteller, 2012; Fatica and Bozzoni, 2014). We predicted *trans*-regulated genes by co-expression analysis based on the expression levels of different lncRNA-mRNA pairs in order to better understand their spatiotemporal transcription patterns. The regulatory network of the four comparisons is shown in Figure 2A. Of the matched pairs, 304 *trans*-regulatory matches were present in the three upper axillary bud pairs, and 214 were co-expressed only in the lower axillary bud pairs (Figure 2B). The 12 hu vs. Cku group had the highest number of matched lncRNA-mRNA pairs (2,243). LncRNAs were co-regulated by multiple mRNAs (up to 225). Overall, 70 (28.9%) lncRNAs had only one *trans*-regulated gene, and 18 (7.4%) were co-expressed with more than 100 target mRNAs (Figure 2C). More than 32% of the mRNAs were co-expressed by single lncRNAs, and 274 (5%) were co-regulated by more than five lncRNAs (Figure 2D).

### Enrichment Analysis of Genes Regulated by Long Non-coding RNAs *via Trans*-Regulatory Activity

To clarify the functions of lncRNAs in response to axillary bud development at different time points and positions, we analyzed the KEGG pathways to identify the *trans*-regulatory genes of lncRNAs in the following comparisons: 3 hu vs. Cku, 12 hu vs. Cku, 24 hu vs. Cku, and 3 hd vs. Ckd. The potential target genes for *trans*-regulation were analyzed for statistical significance using KOBAS platform for KEGG pathway analysis (Supplementary Figure 2). The “plant hormone signal transduction” and “cell cycle” pathways were enriched in all four groups. Furthermore, some glycometabolism pathways—including “starch and sucrose metabolism,” “glycolysis/gluconeogenesis,” and “pyruvate metabolism”—were significantly enriched in this experiment. To verify the reliability of the expression data, we selected six differentially expressed lncRNAs and their target mRNAs involved in plant hormone signal transduction and glycometabolism for qRT-PCR analysis at different developmental stages (Supplementary Figure 3). These results were consistent with the RNA-seq data, and showed that these identified lncRNAs regulated and were co-expressed with genes performing diverse roles in the regulation of axillary buds.

### Long Non-coding RNAs and Their Co-expressed Genes Associated With Hormone Signal Transduction

**FIGURE 3:**
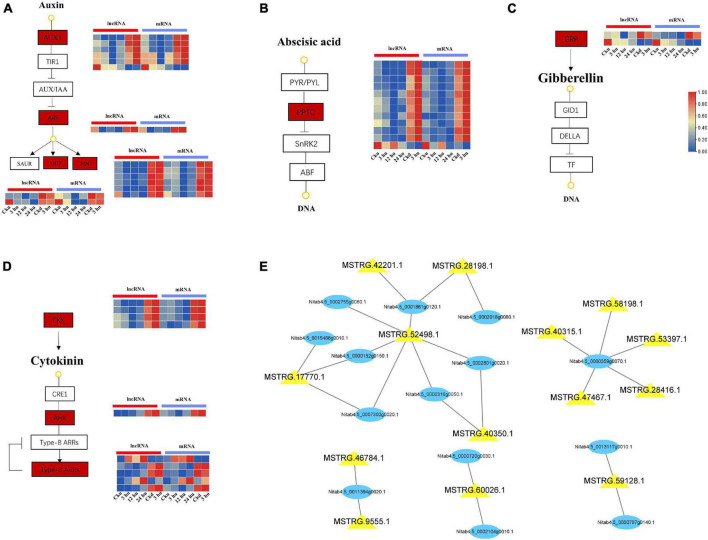
Expression patterns of lncRNAs and mRNAs involved in hormone signal transduction pathways of upper and lower axillary bud. All the heatmaps were generated using Tbtools software with log_2_ transformed FPKM values. **(A)** lncRNAs and mRNAs involved in auxin signal transduction pathway. **(B)** lncRNAs and mRNAs involved in abscisic acid signal transduction pathway. **(C)** lncRNAs and mRNAs involved in cytokinin signal transduction pathway. **(D)** lncRNAs and mRNAs involved in gibberellin signal transduction pathway. The boxes represent mRNAs and lncRNAs. **(E)** mRNA-lncRNA interaction networks in the hormone signal transduction pathways. Blue nodes represent mRNAs, and yellow nodes represent lncRNAs.

Plant hormones have an extremely wide spectrum of activity in the regulation of plant branching development, and the interaction between hormones forms a dynamic balance (Dun et al., 2012). In our study, a total of 27 genes co-expressed with lncRNAs were involved in the “plant hormone signal transduction” pathway (Supplementary Table 4). These included 10 genes associated with IAA, 8 with CTK, 7 with ABA, and 2 with GA. Preliminary co-expression analysis revealed that 10 lncRNAs were highly correlated with genes related to auxins (*AUX1*, *PIN*, *GH3*, and *ARF*) and formed 15 interaction pairs in the axillary buds. The expression levels of *AUX1*, *PIN*, *GH3*, and *ARF* decreased at 12 hu and increased at 24 hu. In addition, MSTRG.52498.1 could *trans*-regulate *AUX1* and *PIN*, and MSTRG.60026.1 could *trans*-regulate *PIN* and *ARF* (Figure 3A). Among the genes associated with ABA, 7 *PP2C* genes were *trans*-regulated by 10 lncRNAs. Among these, MSTRG.52498.1 and MSTRG.17770.1 could regulate two *PP2C* genes (Figure 3B). Nitab4.5_0002950g0060.1 and Nitab4.5_0001642g0050.1 were found to encode gibberellin-regulated protein (GRP), and exhibited a downward expression trend in axillary buds after topping (Figure 3C). The co-expression network of CTK signal transduction contained 12 matched lncRNA-mRNA pairs, including 9 lncRNAs and 8 mRNAs. In particular, two *CKX* genes were targeted by the four lncRNAs and showed a downward expression trend, whereas the *ARR* and *AHK* genes showed increased expression (Figure 3D). The overall in-depth analysis of these plant hormone-related genes and lncRNAs revealed that some lncRNAs could regulate multiple genes simultaneously. MSTRG.52498.1 could *trans*-regulate seven genes encoding IAA, CTK, and ABA; MSTRG.17770.1 could *trans*-regulate three genes belonging to the IAA, CTK, and ABA signal transduction pathways; MSTRG.17770.1 could *trans*-regulate three genes belonging to the IAA, CTK, and ABA signal transduction pathways and MSTRG.60026.1 could *trans*-regulate two genes belonging to the IAA signal transduction pathway (Figure 3E).

### Long Non-coding RNAs and Their Co-expressed Genes Associated With Glycometabolism

**FIGURE 4:**
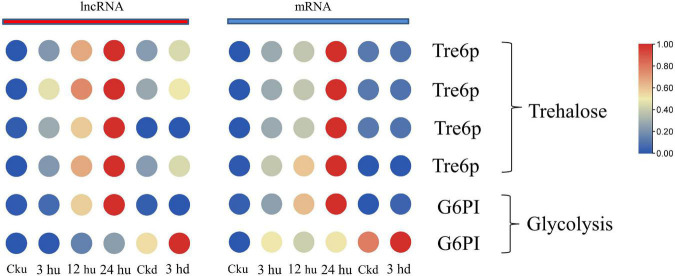
Expression patterns of lncRNAs and mRNAs involved in glycometabolism of upper and lower axillary bud. All the heatmaps were generated using Tbtools software with log_2_ transformed FPKM values.

Axillary bud growth is also determined by the transport of nutrients (such as sugar) and sugar metabolism signals (Liu W. et al., 2019). In our study, we focused on the mRNAs regulated by lncRNAs involving in trehalose metabolism and glycolysis. The expression patterns of mRNA and co-expressed lncRNAs are listed in Supplementary Table 5. The expression of glucose-6-phosphate isomerase (G6PI)—an important enzyme in the glycolytic pathway—was regulated by two lncRNAs and two mRNAs, with two matched lncRNA-mRNA pairs. Two genes encoding G6PI had higher expression levels in the upper axillary buds of the three topped samples than in the un-topped upper axillary buds (Figure 4). As a sucrose signaling metabolite, trehalose 6-phosphate (Tre6P) has been shown to control shoot branching (Fichtner et al., 2017). The expression of Tre6P-related genes was regulated by three lncRNAs that formed four lncRNA-mRNA pairs in the network. Specifically, the expression of Nitab4.5_0001992g0100.1 was simultaneously regulated by three lncRNAs. In addition, MSTRG.32043.1 could also *trans-regulate* two target genes (Figure 4). The lncRNAs involved in sugar metabolism showed a consistent pattern of co-expression with the target genes, indicating that these lncRNAs promoted the expression of the target genes.

### Long Non-coding RNAs and Their Co-expressed Genes Associated With Axillary Bud Regulation

**FIGURE 5:**
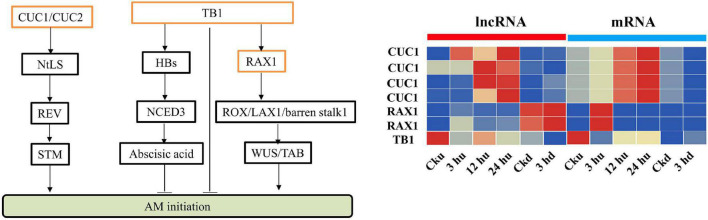
Expression patterns of lncRNAs and mRNAs regulating axillary bud development. All the heatmaps were generated using Tbtools software with log_2_ transformed FPKM values.

Antisense lncRNAs are extensively involved in regulating the expression of protein-coding genes. Many genes involved in the regulation of axillary buds have been identified (Shinohara et al., 2014; Guo et al., 2017; Moreno-Pachon et al., 2018), and the antisense lncRNAs of these genes were analyzed. We identified MSTRG82151.1 as an antisense lncRNA of *NtTB1*, whose expression level was reduced in the axillary buds. In addition, RAX2 was predicted to be trans-regulated by MSTRG.25825.1, and the corresponding lncRNA of *CUC1* was identified as MSTRG.11011.1 (Figure 5). Expression analysis showed that MSTRG.25825.1 and MSTRG.11011.1 exhibited expression trends similar to those of their target genes, suggesting that these lncRNAs could promote the expression levels of their target gene mRNA.

### Expression Profiling of TFs and Their Co-expressed Long Non-coding RNAs in Tobacco

**FIGURE 6:**
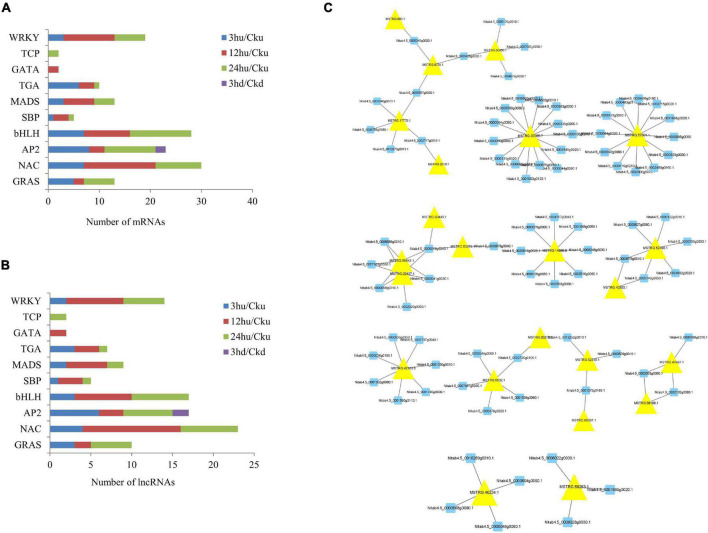
Distribution of transcription factor gene families and their co-expressed lncRNAs in the four treatments (3 hu, 12 hu, 24 hu, and 3 hd vs. the respective controls). **(A)** Distribution of transcription factors in all four treatments. **(B)** Distribution of lncRNAs co-expressed with transcription factors in all four treatments. **(C)** mRNA-lncRNA interaction networks of transcription factors and their co-expressed lncRNAs. Blue nodes represent mRNAs, and yellow nodes represent lncRNAs.

TF regulation is an important part of gene expression and regulation mechanisms in plants, and is involved in the regulation of plant growth and development (Liu M.M. et al., 2019). In our study, eight TFs—including GRAS, NAC, AP2, bHLH, SBP, MADS, TGA, TCP, and WRKY—were found to be co-expressed by lncRNAs. The number of mRNAs and lncRNAs in each group are shown in Figures 6A,B. Two TFs (GATA) were specifically expressed in upper axillary buds at 12 hu, and two TFs (TCP) were specifically expressed in upper axillary buds at 24 hu. Only two AP2 genes were expressed in the lower axillary buds at 3 hd. In the upper axillary buds topped for 3 h, 40 TFs classified in eight TF families were co-expressed with 24 differentially expressed lncRNAs. In the axillary buds topped for 12 h, 44 differentially expressed lncRNAs were found to regulate 52 TFs classified into nine TF families. In upper axillary buds topped for 24 h, 36 differentially expressed lncRNAs were found to regulate 51 TFs classified into nine TF families. In the lower axillary buds topped for 3 h, two differentially expressed lncRNAs regulating two TFs were classified into the AP2 family (Supplementary Table 6). The regulatory networks of lncRNA-mRNA pairs for TFs are shown in Figure 6C. Among these, MSTRG.37035.7 and MSTRG.75790.1 could regulate most genes.

### Hormone Content of Axillary Buds at Different Positions After Topping

**FIGURE 7:**
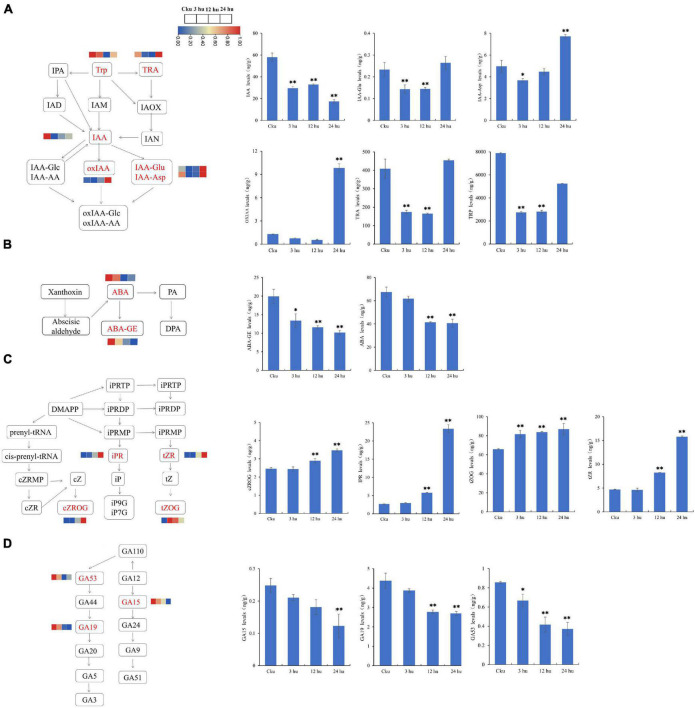
Hormone levels of axillary buds at different positions in un-topped and topped tobacco plants 3, 12, and 24 h after treatment. **(A)** The auxin biosynthetic pathway and the levels of different auxin components. **(B)** The abscisic acid biosynthetic pathway and the levels of different abscisic acid components. **(C)** The cytokinin biosynthetic pathway and the levels of different cytokinin components. **(D)** The gibberellin biosynthetic pathway and the levels of different gibberellin components. The vertical bars indicate standard error (*n* = 3). *P < 0.05, significant correlation; **P < 0.01, extremely significant correlation.

We measured the concentrations of auxin, CTK, GA, and ABA to determine the hormonal variations in topped and un-topped tobacco. We detected six auxin compounds: indole-3-acetic acid (IAA), indole-3-acetyl-L-aspartic acid (IAA-Asp), indole-3-acetyl glutamic acid (IAA-Glu), 2-oxindole-3-acetic acid (OxIAA), tryptamine (TRA), and L-tryptophan (TRP) (Figure 7A). Among these, TRP had the highest concentration, followed by TRA. The concentrations of TRP and TRA decreased from 3 to 12 h and then increased at 24 h. Abscisic acid was detected in the form of abscisic acid (ABA) and ABA-glucosyl ester (ABA-GE), and the concentration of ABA showed a decreasing trend (Figure 7B). We found four cytokinin compounds: *cis*-zeatin-O-glucoside riboside (Czrog), N6-isopentenyladenosine (IPR), *trans*-Zeatin-O-glucoside (tZOG), and *trans*-zeatin riboside (tZR). Cytokinin concentrations increased from 3 to 24 h, and most of the compounds reached peak concentrations at 24 h (Figure 7C). We also detected three GAs: gibberellin A15 (GA15), gibberellin A19 (GA19), and gibberellin A53 (GA53); the GA concentrations showed a decreasing trend in the topped tobacco plants (Figure 7D).

### Functional Validation of Four Differentially Expressed Long Non-coding RNAs in Transgenic Plants

**FIGURE 8:**
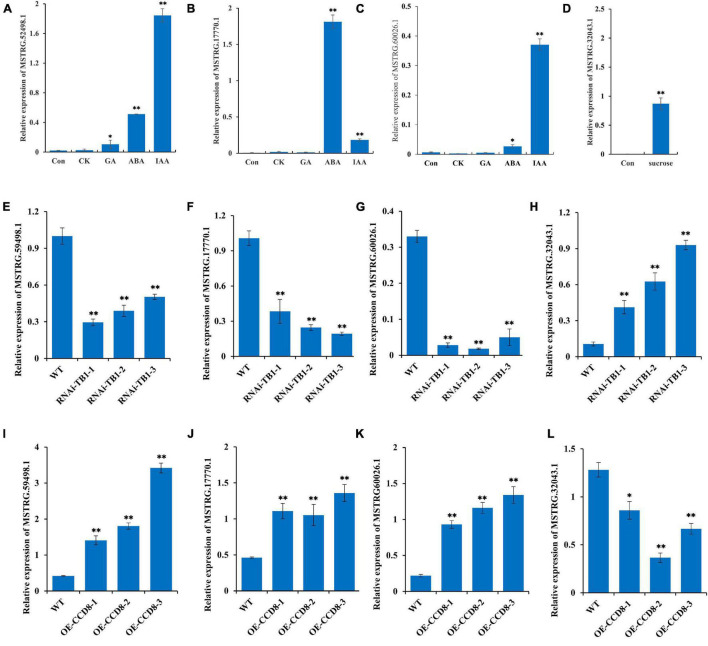
Functional verification of four lncRNAs following treatment with hormones and sucrose in transgenic plants. **(A–C)** Expression levels of MSTRG.52498.1, MSTRG.60026.1, and MSTRG.17770.1 following treatments with different hormones (IAA: indole acetic acid; CTK: cytokinins; ABA: abscisic acid; and GA: gibberellic acid). **(D)** Expression levels of MSTRG.32043.1 following treatment with sucrose. **(E–H)** Expression levels of MSTRG.52498.1, MSTRG.60026.1, MSTRG.17770.1, and MSTRG.32043.1 after RNA interference of *NtTB1* in tobacco plants (*NtTB1*-RNAi). **(I–L)** Expression levels of MSTRG.52498.1, MSTRG.60026.1, MSTRG.17770.1, and MSTRG.32043.1 in tobacco plants with overexpressed *NtCCD8* (*NtCCD8*-OE). The vertical bars indicate SE (*n* = 3). *P < 0.05, significant correlation; **P < 0.01, extremely significant correlation.

In this study, we screened four lncRNAs by co-expression analysis: MSTRG.52498.1, MSTRG.17770.1, MSTRG.60026.1, and MSTRG.32043.1, of which the first three have been implicated in plant hormone transduction. To verify the function of these three lncRNAs, we measured their expression levels under different hormone treatments (IAA, CTK, ABA, and GA). The expression levels of MSTRG.52498.1 and MSTRG.17770.1 were significantly increased under the IAA and ABA treatments and that of MSTRG.60026.1 was significantly increased under the IAA treatment (Figures 8A–C). MSTRG.32043.1 plays an important role in glycometabolism according to our study. Sucrose treatment also enhanced the expression of MSTRG.32043.1, demonstrating that MSTRG.32043.1 has a positive influence on the synthesis of sucrose (Figure 8D).

*TB1* gene has been shown to be a point of integration in the network of various hormones that regulate axillary buds in plants (Gao et al., 2016). Therefore, we performed RNA interference (RNAi) of *NtTB1* in tobacco plants and screened the positive transgenic plants (Supplementary Figure 4A). The relative expression of *NtTB1* was down-regulated by 87.8, 90.7, 70.0%, compared with wild type (Supplementary Figure 4B). Moreover, the RNAi plants promoted the development of axillary buds in untopped tobacco. To further verify the role of these four lncRNAs in axillary bud development, we measured their expression levels in the axillary buds of *NtTB1*–RNAi transgenic tobacco lines. The results showed that the expression levels of MSTRG.52498.1, MSTRG.60026.1, and MSTRG.17770.1 were significantly reduced in the transgenic lines, whereas that of MSTRG.32043.1 was increased (Figures 8E–H). *CCD8* is the synthesis gene of strigolactone, which inhibits axillary bud development. In this study, *NtCCD8* was overexpressed in tobacco plants, and expression levels of *NtCCD8* in transgenic plants were up-regulated (Supplementary Figures 4C,D). The positive transgenic plants showed smaller axillary buds, higher expression levels of MSTRG.52498.1, MSTRG.60026.1, and MSTRG.17770.1, and lower expression levels of MSTRG.32043.1 (Figures 8I–L).

### Functional Validation of MSTRG.28151.1

**FIGURE 9:**
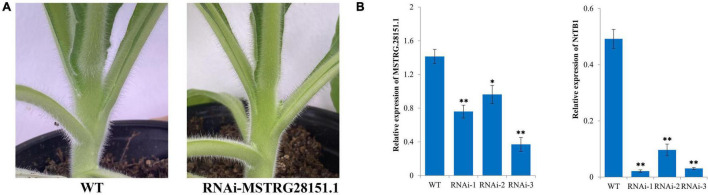
Functional validation of MSTRG.28151.1. **(A)** Phenotype of an axillary bud from MSTRG.28151–RNAi plants. **(B)** Expression levels of *NtTB*1 and MSTRG.28151.1 in the MSTRG.28151–RNAi plants. The vertical bars indicate standard error (*n* = 3). *P < 0.05, significant correlation; **P < 0.01, extremely significant correlation.

Previous studies have shown that *TB1* can be used to control axillary bud development by integrating endogenous and environmental signals (Gao et al., 2016). An antisense lncRNA is a RNA molecule that is transcribed by the antisense chain of a gene (usually a protein-coding gene) and has sequence overlap with the mRNA of the gene (Guil and Esteller, 2012). The antisense lncRNAs of reported axillary bud-related genes have been predicted previously (Fatica and Bozzoni, 2014). In our study, we found that MSTRG.28151.1 could function as an antisense lncRNA of *TB1*. To examine the regulatory effects of the lncRNA on the axillary buds development, a RNAi construct of MSTRG.28151.1 was created and transformed into tobacco plants. Three independent MSTRG.28151.1-RNAi lines were obtained. Phenotype of untopped plants shown the larger axillary buds in the MSTRG.28151.1-RNAi plants, compared to the wild type (Figure 9A). Analysis of the transcript levels indicated the downregulation level of MSTRG.28151.1 and *NtTB1* (Figure 9B).

## Discussion

### Topping Changes the Transcription Level of Long Non-coding RNAs in Axillary Buds

Research on the regulation of branch growth in plants is important for improving crop yield and quality (Teichmann and Muhr, 2015; Mathan et al., 2016). Apical dominance increases the preference for terminal bud growth and inhibits the growth of lateral branches in many plants (Barbier et al., 2017). Several reactions occur in tobacco after topping, including rapid the axillary bud growth rapidly, which are regulated by hormones, sugar and other complex internal factors (Xia et al., 2019). Previous studies have shown that lncRNAs participate in the regulation of plant growth and development, seed germination, reproductive development, flowering time regulation, hormone signaling and stress tolerance (Liu F. et al., 2019; Yang et al., 2019; Yu et al., 2019; Ramírez Gonzales et al., 2020). However, we did not find any previous literature regarding the role of lncRNAs in axillary bud development. In this study, germination and elongation of axillary buds were promoted after topping in tobacco, and the upper axillary buds germinated earlier than the lower axillary buds. To better understand and clarify the response of lncRNA levels in axillary buds after topping, transcriptome sequencing and annotation were performed on both untopped and topped axillary buds. Most lncRNAs were < 1,000 nucleotides in length and contained only 1–2 exons, similar to the lncRNAs of other plants (Yan et al., 2020; Huang et al., 2021). Some studies have found that the functions of lncRNAs are highly correlated with time. In our study, we observed phenotypic changes in axillary buds at three time points in topped and un-topped tobacco. Some of the identified lncRNAs were differentially expressed at a single time point, whereas some were differentially expressed at multiple time points. Differential expression of lncRNAs at different times may regulate axillary bud development in a more dynamic manner. Furthermore, the number of differentially expressed lncRNAs in the upper axillary buds was greater than in the lower axillary buds after topping. Thus, when apical dominance was eliminated after topping, the response was prioritized in the upper axillary buds of tobacco plants. Previous studies have shown that different axillary buds compete for the auxin transport channel from the main stem to the roots, which leads to a decrease in polar auxin transport capacity (Ljung et al., 2010; Francois et al., 2019). In our study, the activated axillary buds located in the upper part of the plant may have inhibited the ability of other axillary buds to export auxin. Moreover, we systematically measured the hormonal titers in the upper axillary buds of the four groups to understand the physiological mechanisms of hormone-regulated axillary bud development. By comparing the results between groups of un-topped and topped tobacco plants, we found that topping reduced the levels of different types of GAs (GA15, GA19, and GA53) and ABA components (ABA and ABA-GE). This indicates that GA and ABA can inhibit the development of axillary buds, which is consistent with previously reported results (Knox and Wareing, 1984; Yao and Finlayson, 2015; Zhuang et al., 2019; Zhang et al., 2020). The concentrations of different types of auxins decrease initially and then increased, whereas CTK concentrations showed the opposite trend. This suggests that auxins and CTK coordinate the dormancy and outgrowth of axillary buds (Qiu et al., 2019; Zhao et al., 2020).

### Long Non-coding RNAs Regulate Plant Hormones to Affect Axillary Bud Development

Many hormones, including IAA, GA, ABA, and CTK are involved in the regulation of axillary bud development (Phillips, 1971; Chatfield et al., 2000; Wen et al., 2020; Yan et al., 2021). In this study, we simultaneously analyzed the genes related to hormone transduction and measured hormone titers, which allowed us to elucidate the mechanism of hormone regulation in axillary buds. The expression levels of *AUX1, PIN, GH3*, and *ARF*—genes associated with IAA transduction—changed significantly after topping, and we identified 10 lncRNAs capable of *trans*-regulating their expression. The expression of *PIN* was reduced in the 3hu_Cku and 12hu_Cku groups, and increased in the 24hu_Cku group. This was similar to results reported in *Arabidopsis* indicating that IAA can control the meristem of the axillary bud *via* direct regulation of the *PIN* gene (Balla et al., 2011). The expression of some lncRNAs has been reported to correlate with that of IAA. For example, some lncRNAs were found to respond to salt stress *via* the IAA-mediated pathway in poplars (Ma et al., 2019). In our study, MSTRG.52498.1 could *trans*-regulate the expression of three genes, including *AUX1* and *PIN*, which were all significantly downregulated at 3 and 12 h after topping, indicating that auxin can inhibit the activation of dormant axillary buds. This suggested that such lncRNAs were involved in the regulation of cytokinin, and cannot only activated the dormant axillary buds, but also promote the growth of axillary buds. The expression levels of *AUX1* and *PIN* were correlated with auxin concentrations; therefore, we predicted that MSTRG.52498.1 may influence auxin concentrations by regulating the expression of *AUX1* and *PIN*. CTK, a class of phytohormones, are generally found in the stem tip, root tip, bud tip, and meristem, and promote cell division and differentiation (Gasparis et al., 2019). *CKX*, *AHK*, and *ARR* are the key genes controlling the concentration of CTK (Wang et al., 2014; Gasparis et al., 2019). In our study, the most highly expressed lncRNA (MSTRG.19643.2) was co-expressed with *ARR* (fold change of 4.8, 4.6, and 5.5 at 3, 12, and 24 h, respectively) in three samples of axillary buds. The expression of Nitab4.5_0001861g0120.1 (encoding CKX) decreased significantly, and it was also co-expressed with MSTRG.52498.1, MSTRG.28198.1, and MSTRG.42201.1. The downregulation of *CKX* was consistent with the findings of axillary bud outgrowth in apples (Tan et al., 2018). It may be expected that MSTRG.52498.1, MSTRG.28198.1, and MSTRG.42201.1 increase the levels of Czrog, IPR, tZOG, and tZR, thus inhibiting the expression of CKX. ABA is another major plant hormone that plays a key role in plant responses to abiotic and biological stresses and inhibits seed germination and lateral branch growth (Holalu et al., 2020). PP2C is the second messenger in the ABA signal transduction pathway. In our study, the expression of 20 *PP2C* genes encoding ABA receptors decreased significantly after topping, and 22 lncRNAs were predicted to regulate their expression. The downregulation of *PP2C* genes was consistent with the results of a previous study in rice, where PP2C genes were shown to control the stem cell expansion of axillary buds (Miao et al., 2020). Among the lncRNAs involved, MSTRG.52498.1 had the highest number of target genes. The lncRNA expression profiles were consistent with those of previous studies, confirming the importance of lncRNAs in the signal transduction of ABA (Shumayla et al., 2017; Wang Z. et al., 2018). The concentrations of ABA and ABA-GE were reduced, indicating that MSTRG.52498.1 and nine other lncRNAs regulated the expression of *PP2C* genes by regulating the levels of ABA. In addition, the expression levels of some lncRNAs were decreased at different time points, indicating that these lncRNAs play a certain role in axillary bud germination and elongation. For instance, MSTRG.1182.158253.1 MSTRG. GA is involved in every stage of plant growth and development and also affects the physiological indexes of plant growth and development (Ni et al., 2015). GA can affect internode elongation and alter cell wall biosynthesis. *GRP* is a key gene in GA signal transduction that regulates the growth and development of rice (Beatty et al., 2009). The expression level of *GRP* gene regulated by MSTRG.32988.1 was decreased significantly in the three groups, similar to the titers of GA15, GA19, and GA53. This was consistent with results of a study on the hybrid aspen (Abe et al., 2014). This suggests that MSTRG.56740.1 and MSTRG.32988.1 may play an inhibitory role in regulating axillary bud growth by regulating GA15, GA19, and GA53. After a holistic analysis of plant hormone-related genes and lncRNAs, we found that MSTRG.52498.1 could regulate a variety of hormones, including IAA, CTK, and ABA, whereas MSTRG.17770.1, MSTRG.28198.1, and MSTRG.32988.1 could regulate two hormones. The effects of different hormone treatments on lncRNA expression levels confirmed the reliability of our results and indicate that these techniques can be used for further research and exploration.

### Long Non-coding RNAs Regulate Glycometabolism to Affect Axillary Bud Development

Sucrose is an important signaling molecule involved in the regulation of branching development in plants (Barbier et al., 2015; Kebrom and Mullet, 2015). As an energy source, sucrose can directly affect the growth of axillary buds. However, it also interacts with CTK, IAA, and SLs to promote the development of axillary buds (Mishra et al., 2009; Rabot et al., 2012; Bertheloot et al., 2020). To identify the key lncRNAs affecting axillary bud development, we performed an in-depth co-expression analysis for lncRNAs enriched in glycolysis and their target genes. Glycolysis is a basic pathway for energy metabolism in plants involving glucose degradation. Previous studies have shown that glycolysis regulates polar-specific regulation of pollen tube growth in plant cells (Wang et al., 2020). In our study, the expression of *G6PI* was significantly upregulated in the upper and lower axillary buds, suggesting that *G6PI* plays an important role in the development of axillary buds and the activation of dormant axillary buds. Tre6P is a sucrose-signaling metabolite that controls shoot branching (Fichtner et al., 2017; Fichtner and Lunn, 2021). In this study, we found that two genes encoding Tre6P responded positively to the development of axillary buds and were co-expressed with MSTRG.32043.2, MSTRG.58801.1, and MSTRG.32043.1. These results are consistent with those of previous studies on the garden pea, suggesting that MSTRG.32043.2, MSTRG.58801.1, and MSTRG.32043.1 may have far-reaching effects on axillary buds and shoot branching. In conclusion, we found evidence of a link between sugar metabolism and axillary bud development after topping. Our findings regarding the expression levels of lncRNAs after sucrose treatment help us advance our understanding of the mechanisms underlying the regulatory role of lncRNAs in axillary bud development.

### Transgenic Plants Verify the Involvement of Long Non-coding RNAs in Axillary Bud Development

In this study, three lncRNAs—MSTRG.52498.1, MSTRG.17770.1, and MSTRG.60026.1—were co-expressed with hormone-related genes. Experiments with different hormone treatment further proved that these lncRNAs were indeed induced by hormones. MSTRG.32043.1 was co-expressed with sucrose-related genes, and was stimulated by sucrose treatment. These results are consistent with a study on peanut seed development, which reported that lncRNAs participated in plant growth and development by regulating hormones (Ma et al., 2020). *CCD8* and *TB1* are vital genes that are known to regulate axillary bud development (Dong et al., 2017; Wang X. et al., 2021). In our preliminary experiment, the plants with overexpressed *NtCCD8* had fewer axillary buds, whereas the plants with RNAi of *NtTB1* had more axillary buds. In the two transgenic lines, the expression levels of MSTRG.52498.1, MSTRG.17770.1, MSTRG.60026.1, and MSTRG.32043.1 showed the same trends as those in the topped treatments. This further indicated that lncRNAs influence axillary bud development by regulating hormones and sucrose.

Antisense lncRNAs are mainly produced by the antisense chain of mRNAs. By analyzing the genes reported to regulate axillary buds, we predicted certain antisense lncRNAs in our study. Among these, MSTRG.28151.1 was predicted to be the antisense lncRNA of *NtTB1*, which could be the bud-specific gene inhibiting axillary buds growth (Ding et al., 2020). To further verify the regulatory effect of this lncRNA on *NtTB1*, we constructed a RNAi vector of MSTRG.28151.1. Following RNAi of MSTRG.28151.1, the expression of the *NtTB1* gene was significantly reduced, and the axillary buds of the RNAi transgenic lines were larger than those of the wild type. This indicates that MSTRG.28151.1 could regulate the development of axillary buds. However, the underlying mechanism of this regulation requires further exploration. In addition, some genes, such as *RAX2*, *CUC1*, and *TB1* are known to directly affect the development of axillary buds. *RAX2* regulates the formation of axillary meristems in *Arabidopsis* (Moreno-Pachon et al., 2018), and *CUC1* participates in initiating the shoot apical meristem and establishing organ boundaries (Shinohara et al., 2014). During co-expression, the transcripts of *RAX2* were targeted by MSTRG.12195.1, whereas the transcripts of *CUC1* were targeted by MSTRG.25624.1 and MSTRG.16154.1. The lncRNA-mediated regulation of *RAX2* and *CUC1* expression is consistent with previous reports (Hibara et al., 2003; Yang et al., 2012; Aida et al., 2020; Jia et al., 2020). This demonstrates that MSTRG.12195.1, MSTRG.25624.1, and MSTRG.16154.1 are key lncRNAs in axillary bud development. These findings of lncRNAs expand our knowledge of the possible roles of lncRNAs in axillary bud development.

## Conclusion

In this study, we performed a dynamic lncRNA-seq of tobacco during different stages of axillary bud development. Topping promoted axillary bud development due to changes in the expression levels of lncRNAs associated with hormone signal transduction and glycometabolism, compared to that of untopped plants. Topping regulated the transcription of TFs, such as TCP, NAC, AP2, and GRAS, which can directly regulate the development of axillary buds. Functional validation of few lncRNAs implicated in hormone transduction and glycometabolism, confirmed the role of lncRNAs in axillary bud development. Moreover, RNA interference of MSTRG.28151.1 in tobacco plants induced axillary bud outgrowth, which proved the direct role of lncRNA in axillary bud development. In summary, the results of this study not only improve our understanding of plant responses and adaptations for axillary bud development, but also reveal that the lncRNAs play a critical role in controlling the axillary bud growth in plants. Nevertheless, the molecular mechanism of lncRNAs involved in the regulation of axillary bud development requires further exploration.

## Data Availability Statement

The original contributions presented in the study are publicly available. This data can be found here: the raw data can be downloaded from the ftp site of China National GeneBank DataBase (CNGBdb), using the link (https://ftp.cngb.org/pub/CNSA/data3/CNP0002382).

## Author Contributions

LW, JG, and XX conceived the experiments. LW drafted the manuscript. CW and XL participated in the main experiments in this work, with assistance from JY, KC, and YK. YX and PC processed and analyzed the data. YW revised the scientific content of the manuscript. All authors contributed to the article and approved the submitted version.

## Conflict of Interest

JG, XL, KC, and YW were employed by China Tobacco Hunan Industrial Co., Ltd. The remaining authors declare that the research was conducted in the absence of any commercial or financial relationships that could be construed as a potential conflict of interest.

## Publisher’s Note

All claims expressed in this article are solely those of the authors and do not necessarily represent those of their affiliated organizations, or those of the publisher, the editors and the reviewers. Any product that may be evaluated in this article, or claim that may be made by its manufacturer, is not guaranteed or endorsed by the publisher.
