# Supplementary figures and images for: Comprehensive Analysis of Long Non-coding RNA Modulates Axillary Bud Development in Tobacco (Nicotiana tabacum L.)
Source: Front Plant Sci. 2022 Feb 14;13:809435. doi: 10.3389/fpls.2022.809435 (PMC8884251; doi:10.3389/fpls.2022.809435)

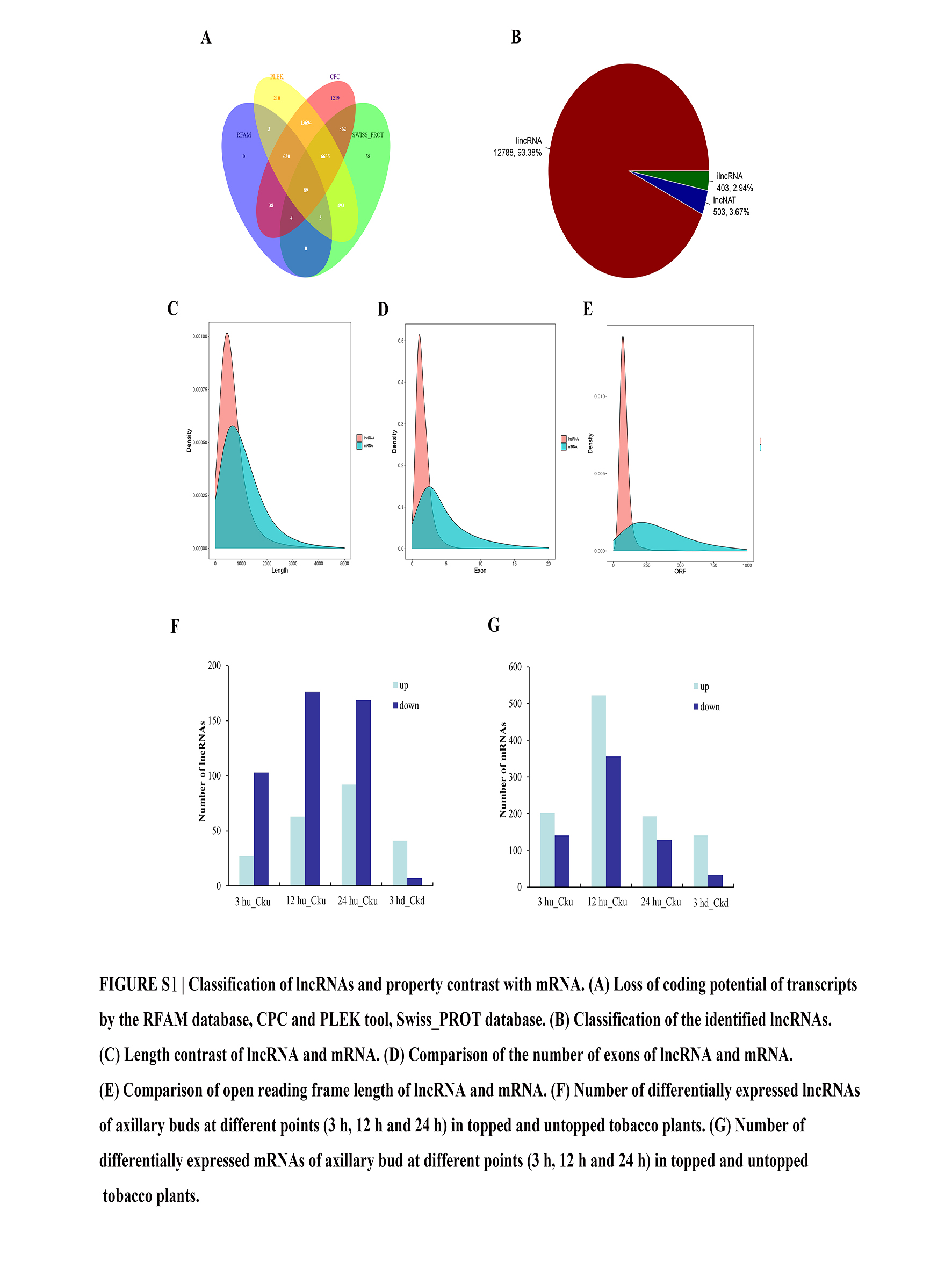

Supplement: Supplementary file 1 [file Image_1.JPEG]

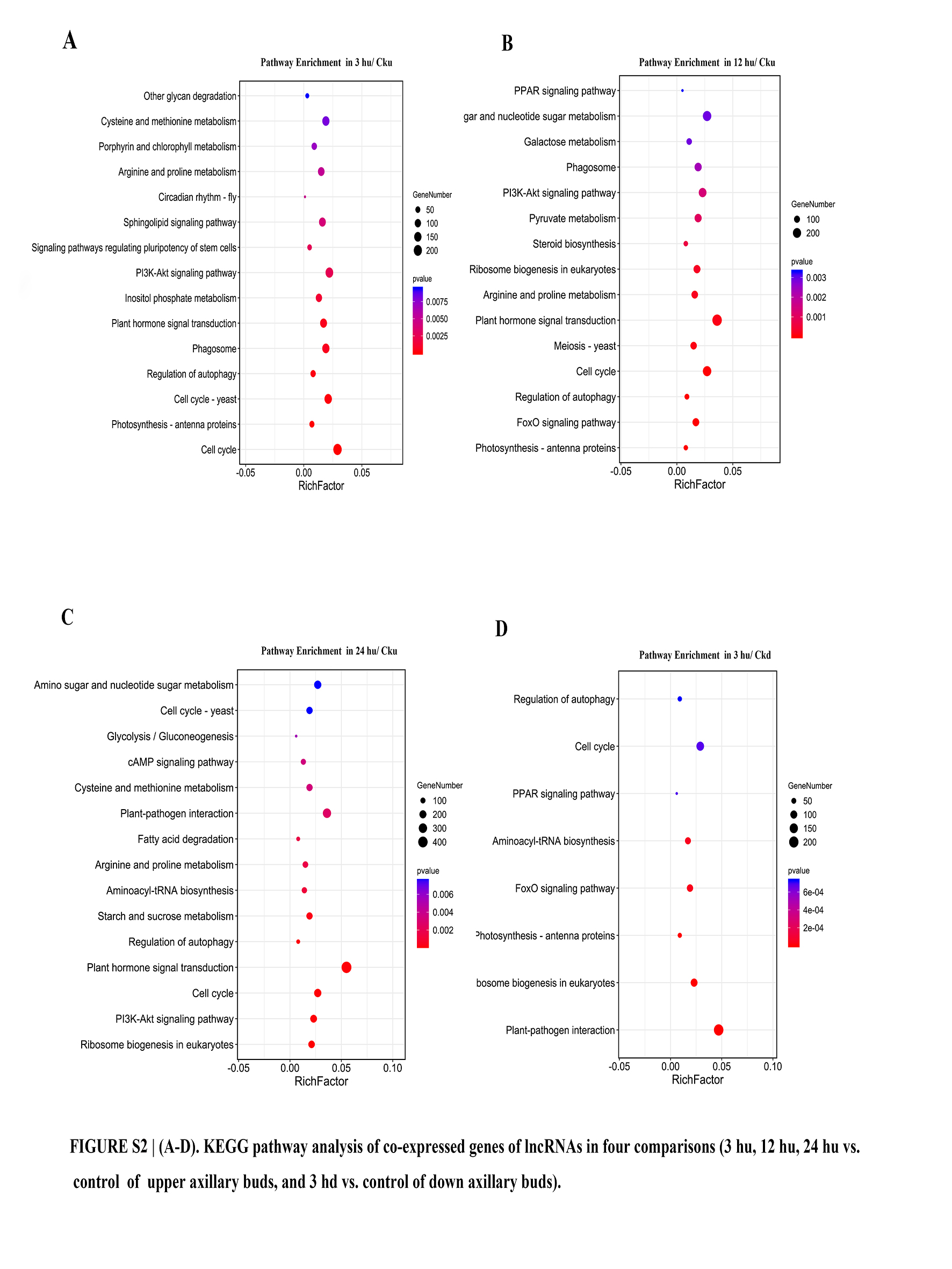

Supplement: Supplementary file 2 [file Image_2.JPEG]

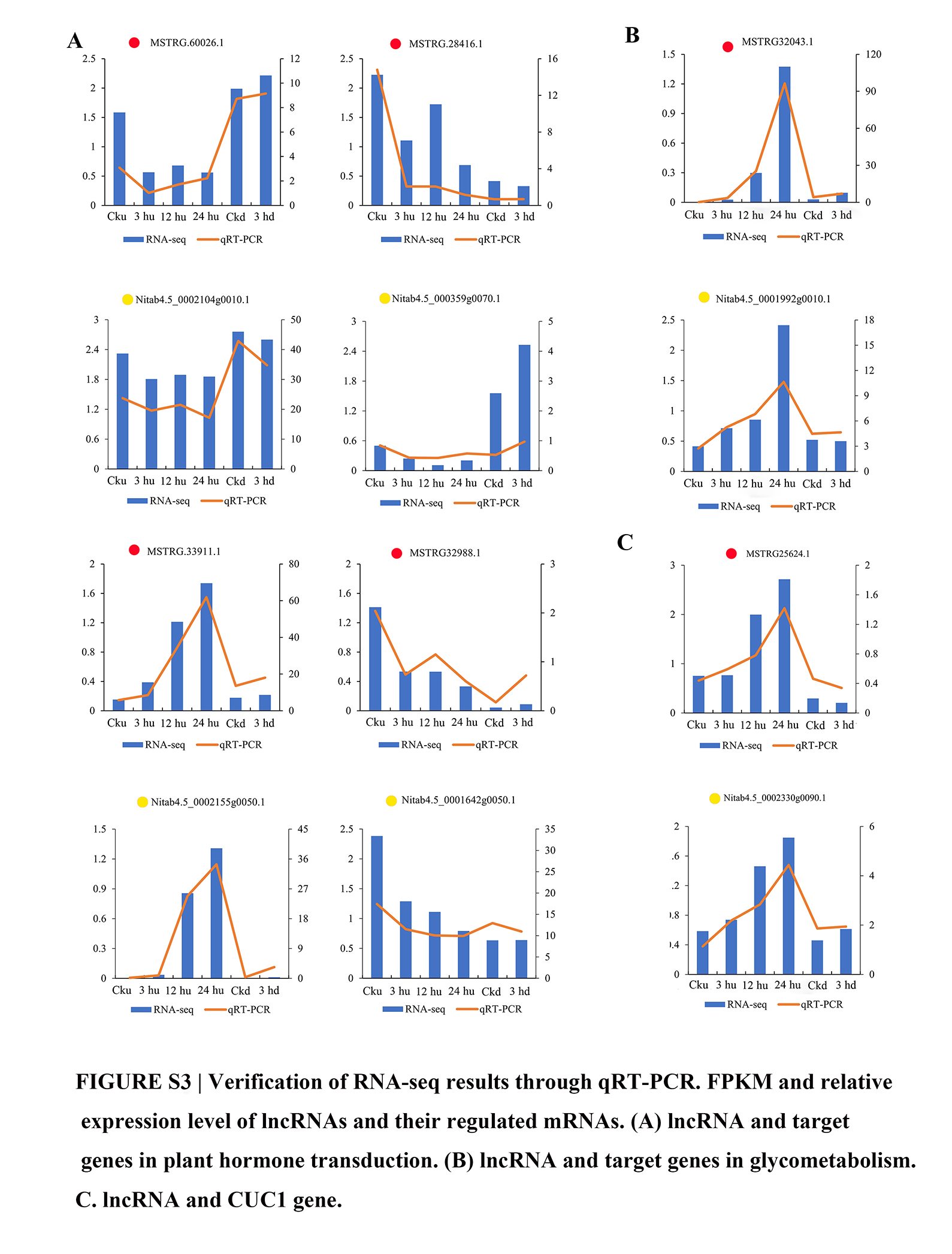

Supplement: Supplementary file 3 [file Image_3.JPEG]

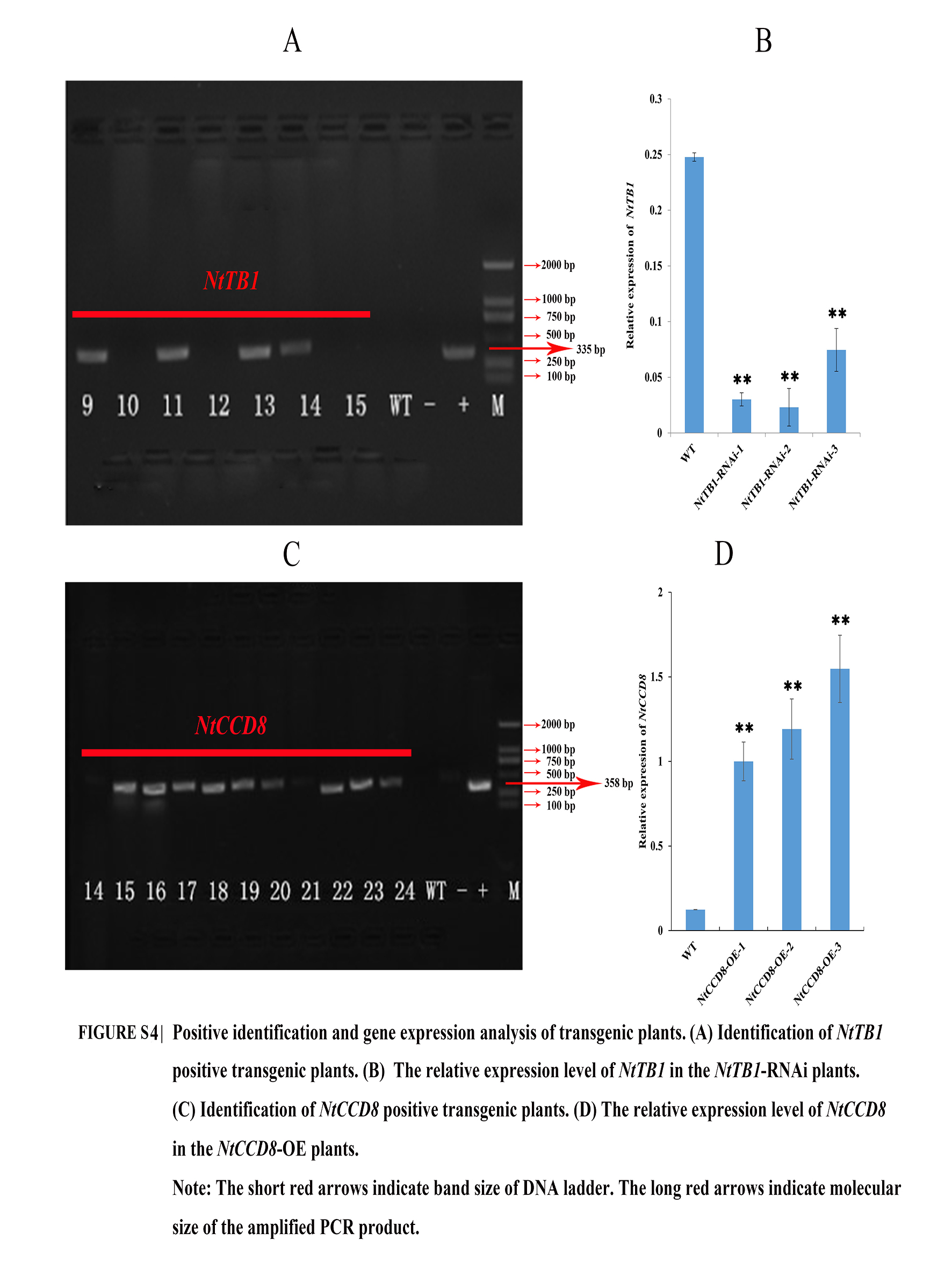

Supplement: Supplementary file 4 [file Image_4.JPEG]
